# Self-reported general health, physical distress, mental distress, and activity limitation by US county, 1995-2012

**DOI:** 10.1186/s12963-017-0133-5

**Published:** 2017-04-26

**Authors:** Laura Dwyer-Lindgren, Johan P. Mackenbach, Frank J. van Lenthe, Ali H. Mokdad

**Affiliations:** 1 Institute for Health Metrics and Evaluation, University of Washington, 2301 5th Ave, Suite 600, Seattle, WA 98121 USA; 2 Department of Public Health, Erasmus MC, Rotterdam, Netherlands

**Keywords:** Disparities, Inequalities, Small area estimation, Self-reported health, Health-related quality of life, Healthy Days, Behavioral Risk Factor Surveillance System

## Abstract

**Background:**

Metrics based on self-reports of health status have been proposed for tracking population health and making comparisons among different populations. While these metrics have been used in the US to explore disparities by sex, race/ethnicity, and socioeconomic position, less is known about how self-reported health varies geographically. This study aimed to describe county-level trends in the prevalence of poor self-reported health and to assess the face validity of these estimates.

**Methods:**

We applied validated small area estimation methods to Behavioral Risk Factor Surveillance System data to estimate annual county-level prevalence of four measures of poor self-reported health (low general health, frequent physical distress, frequent mental distress, and frequent activity limitation) from 1995 and 2012. We compared these measures of poor self-reported health to other population health indicators, including risk factor prevalence (smoking, physical inactivity, and obesity), chronic condition prevalence (hypertension and diabetes), and life expectancy.

**Results:**

We found substantial geographic disparities in poor self-reported health. Counties in parts of South Dakota, eastern Kentucky and western West Virginia, along the Texas-Mexico border, along the southern half of the Mississippi river, and in southern Alabama generally experienced the highest levels of poor self-reported health. At the county level, there was a strong positive correlation among the four measures of poor self-reported health and between the prevalence of poor self-reported health and the prevalence of risk factors and chronic conditions. There was a strong negative correlation between prevalence of poor self-reported health and life expectancy. Nonetheless, counties with similar levels of poor self-reported health experienced life expectancies that varied by several years. Changes over time in life expectancy were only weakly correlated with changes in the prevalence of poor self-reported health.

**Conclusions:**

This analysis adds to the growing body of literature documenting large geographic disparities in health outcomes in the United States. Health metrics based on self-reports of health status can and should be used to complement other measures of population health, such as life expectancy, to identify high need areas, efficiently allocate resources, and monitor geographic disparities.

**Electronic supplementary material:**

The online version of this article (doi:10.1186/s12963-017-0133-5) contains supplementary material, which is available to authorized users.

## Background

Measures of survival, such as life expectancy, have long been used to compare the health status of different populations and to track changes in health status over time [1, 2]. While objective, and relatively easily measured, these metrics fail to capture differences in health due to non-fatal (or not yet fatal) conditions [3]. Moreover, they fail to take into account individuals’ own assessment of and satisfaction with their health and functioning. In response to these limitations, metrics based on self-reported health status have been proposed as a complement to objective measures for use in tracking levels of population health over time and for evaluating disparities in health [4, 5].

The Behavioral Risk Factor Surveillance System (BRFSS) is an annual telephone survey conducted in all states and supported by the Centers for Disease Control and Prevention [6, 7]. Since 1993, the BRFSS has included four core “Healthy Days” questions in which respondents are asked to rate their overall health and to report the number of days in the past month that they experienced poor physical health, poor mental and emotional health, or were unable to participate in their usual activities. These questions are designed to elicit respondents’ self-assessment of and satisfaction with their health generally and with their recent physical health, mental and emotional health, and functional limitations [4]. Health metrics based on these and similar questions have been shown to be highly correlated with metrics based on lengthier survey instruments [8, 9], health behaviors and risk factors [10–12], chronic health conditions [13], health care utilization [14], and mortality risk [15].

Healthy days questions from the BRFSS have been used to track national- and state-level trends in poor self-reported health [16] and to explore disparities by gender [17], race/ethnicity [18], socioeconomic status [16], and employment status [19]. However, these data have only been used in a limited way to explore local-level variations in poor self-reported health. Jia et al. [20] and others [21] considered county-level measures of poor self-reported health based on the healthy days questions, but focused on county-level correlates of poor self-reported health rather than on spatial patterns and disparities. The County Health Rankings & Roadmaps Program includes three county-level measures of poor self-reported health based on BRFSS health days questions, but recent methodological changes make it difficult to track trends over time [22].

In this analysis, we used validated small area estimation methods to estimate the prevalence of four measures of poor self-reported health—low general health, frequent physical distress, frequent mental distress, and frequent activity limitation—by county from 1995 to 2012. We used these estimates to explore spatial patterns in poor self-reported health and to quantify county-level geographic disparities.

We performed two additional analyses combining these estimates of poor self-reported health with other estimates of health risk factors and outcomes at the county level. First, we compared the prevalence of poor self-reported health to the prevalence of behavioral and metabolic risk factors (i.e., obesity, smoking, and physical inactivity) and chronic conditions (i.e., hypertension and diabetes) to assess the face validity of self-reported health as a proxy for a county’s population health. We expected the prevalence of poor self-reported health to be higher in places with higher prevalence of risk factors and chronic conditions known to result in considerable health burden.

Second, we compared the prevalence of poor self-reported health to estimates of life expectancy at the county-level, in order to assess whether geographic county-level disparities in life expectancy and self-reported health follow the same pattern. We expected the prevalence of poor self-reported health to be strongly and negatively correlated with life expectancy, albeit not perfectly as life expectancy and self-reported health may reflect somewhat different aspects of a county’s health burden.

## Methods

### Data

We analyzed data from the BRFSS surveys conducted from 1995, the first year in which all 50 states participated in the BRFSS, through 2012, the most recent year in which county identifiers were publicly available. The BRFSS included four “healthy days” questions:

1. Would you say in general that your health is—excellent, very good, good, fair, or poor?
2. Now thinking about your physical health, which includes physical illness and injury, for how many days during the past 30 days was your physical health not good?
3. Now thinking about your mental health, which includes stress, depression, and problems with emotions, for how many days during the past 30 days was your mental health not good?
4. During the past 30 days, for about how many days did poor physical or mental health keep you from doing your usual activities, such as self-care, work, or recreation?

Only the first question was asked by all states in 2002; consequently we excluded data on the remaining questions for this year only.

We created four binary variables from these questions: low general health (responding “fair” or “poor” to question 1); frequent physical distress (reporting 14 or more days in response to question 2); frequent mental distress (reporting 14 or more days in response to question 3); and frequent activity limitation (reporting 14 or more days in response to question 4). The 14 day cut-off used for frequent physical distress, mental distress, and activity limitation is in line with previous research utilizing these questions, and is intended to identify individuals who experienced significant health burden in the previous month [10–12, 17–19]. In addition, we extracted county of residence, age, gender, race/ethnicity (white non-Hispanic, black non-Hispanic, native non-Hispanic, or Hispanic), education status (less than high school, high school graduate, some college, or college graduate), marital status (never married, currently married, or formerly married), and, starting in 2011, phone type (landline only, cell phone only, or dual) from the survey. Respondents with missing values on any of these variables were excluded from the analysis. There were 5,239,833 respondents in the study period. Of these, 2.2% were missing some demographic information, 3.8% were missing one or more outcome variables, and 5.1% were missing the county variable, primarily due to CDC data suppression rules. In total, 4,698,203 (89.7%) had no missing values and were included in the analysis. The survey response rate in the BRFSS varied by year and by state; in 2012, the response rate ranged from 27.7 to 60.4% among states [23].

### Small area estimation model

We used previously described and validated small area models to estimate county-level prevalence of low general health, frequent physical distress, frequent mental distress, and frequent activity limitation [24]. These models are designed to “borrow strength” across time, space, and from external data sources (i.e., covariates) in order to increase the effective amount of information available for each county. Briefly, these models were specified as:

$$ \begin{array}{c}\hfill {Y}_{j, t, a, r, m, e}\sim \mathrm{Binomial}\left({p}_{j, t, a, r, m, e},\ {N}_{j, t, a, r, m, e}\right)\hfill \\ {}\hfill \mathrm{logit}\left({p}_{j, t, a, r, m, e}\right) = {\beta}_0+{\beta}_{1, a}+{\beta}_{2, r}+{\beta}_{3, m} + {\beta}_{4, e}+{\boldsymbol{\beta}}_{\mathbf{5}}\cdot {\boldsymbol{X}}_{\boldsymbol{j},\boldsymbol{t}}+{u}_j+{w}_t+{d}_{j, t}\hfill \end{array} $$

where *N*_*j*,*t*,*a*,*r*,*m*,*e*_, *Y*_*j*,*t*,*a*,*r*,*m*,*e*_, and *p*_*j*,*t*,*a*,*r*,*m*,*e*_ are the total number of respondents; the number of respondents with low general health, frequent physical distress, frequent mental distress, or frequent activity limitation, depending on the model; and the true prevalence, respectively, in county *j*, year *t*, age group *a*, race/ethnicity group *r*, marital status group *m*, and education group *e*. The *β* terms are fixed effects: *β*_0_ is the intercept; *β*_1,*a*_ are age group effects and are included to account for differences in self-reported health among age groups; *β*_2,*r*_, *β*_3,*m*_, and *β*_4,*e*_ are race/ethnicity, marital status, and education effects, respectively, and are included to account for differences in self-reported health among each of these groups; ***β***_**5**_ is a vector of coefficients on three county-level covariates that are expected to be predictive of poor self-reported health (percent of the population living in poverty, the unemployment rate, and the percent of households which are rural). The remaining terms are random effects. *u*_*j*_ and *w*_*t*_ are county- and year-level random effects, respectively, each of which is assumed to follow a conditional autoregressive distribution that allows for spatial (*u*_*j*_) and temporal (*w*_*t*_) smoothing (specifically, the distribution described by Leroux et al. [25]). *d*_*j*,*t*_ is a county-year-level random effect with a non-separable “Type IV” interaction between space and time as described by Knorr-Held [26], but using the conditional autoregressive distribution described by Leroux et al. [25] for both the spatial and temporal dimensions. Gamma(1, 1000) priors were assigned for the precision parameters of each random effect. Normal(0, 1.5) priors were assigned for the logit-transformed autocorrelation parameter of each random effect.

Models were fit using the TMB package [27] in R version 3.2.4 [28] and 1000 draws of *p*_*j*,*t*,*a*,*r*,*m*,*e*_ were simulated from the posterior distribution. These draws were post-stratified by race, marital status, and education using population counts from the census and American Community Survey to ensure that prevalence estimates represent the demographic composition of a county even where response rates vary among different demographic groups. Draws were then age-standardized using the 2010 census population as the standard. Point estimates were calculated from the mean of the 1000 draws and 95% uncertainty intervals (UIs) were calculated from the 2.5th and 97.5th percentiles. State- and national-level estimates were generated by population-weighting the county-level estimates.

Separate models were fit for males and females for each of the four measures, for eight total models. Prior to 2011, the BRFSS sample did not include cell phones, raising the possibility of non-coverage bias; the correction method described by Dwyer-Lindgren et al. was applied to address this issue [29].

### Comparison to risk factors, chronic conditions, and life expectancy

After modeling county-level prevalence of low general health, frequent physical distress, frequent mental distress, and frequent activity limitation, we compared these measures to existing estimates of county-level prevalence of behavioral and metabolic risk factors (smoking, obesity, and physical inactivity), and chronic conditions (hypertension and diabetes), also derived from BRFSS data [24, 29–31]. For each of these variables, we calculated the Pearson correlation coefficient with each of the four measures of poor self-reported health in the most recent year of data available (ranging from 2009 for hypertension to 2012 for diabetes).

We also compared the prevalence of low general health, frequent physical distress, frequent mental distress, and frequent activity limitation with life expectancy in 2012 (Laura Dwyer-Lindgren, Amelia Bertozzi-Villa, Rebecca W Stubbs, Chloe Morozoff, Johan P Mackenbach, Frank J van Lenthe, Ali H Mokdad, and Christopher JL Murray: Inequalities in life expectancy among US counties, 1980 to 2014: Temporal trends and key drivers., forthcoming). We used loess regression—a non-parametric smoothing technique [32]—to characterize the relationship between each of these four variables and life expectancy. We also examined the correlation between change in prevalence of low general health, frequent physical distress, frequent mental distress, and frequent activity limitation, and change in life expectancy between 1995 and 2012.

## Results

**Fig. 1:**
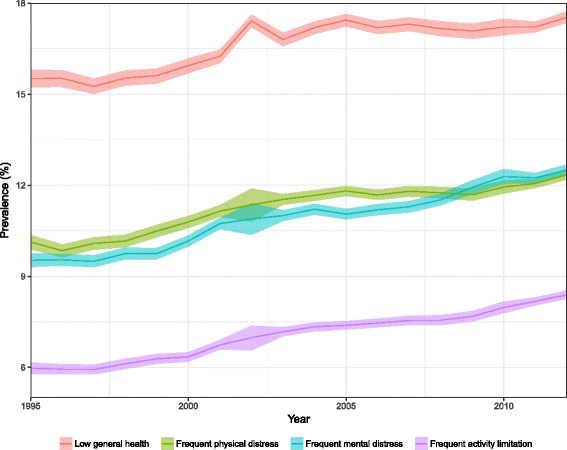
National trends in low general health, frequent physical distress, frequent mental distress, and frequent activity limitation, 1995–2012

Nationally, the prevalence of all four measures of poor self-reported health increased between 1995 and 2012: from 15.5% (95% UI: 15.2–15.8%) to 17.5% (17.4–17.7%) for low general health; from 10.1% (9.9–10.4%) to 12.3% (12.2–12.5%) for frequent physical distress; from 9.5% (9.3–9.7%) to 12.5% (12.3–12.7%) for frequent mental distress; and from 6.0% (5.8–6.2%) to 8.4% (8.3–8.5%) for frequent activity limitation (Fig. 1). The prevalence was higher among women than among men in all years. In 2012, the prevalence among women exceeded that among men by 7.6% (18.2% [18.0–18.4%] vs. 16.9% [16.6–17.1%]) for low general health; 23.2% (13.6% [13.4–13.8%] vs. 11.0% [10.9–11.2%]) for frequent physical distress; 38.6% (14.5% [14.3–14.6%] vs. 10.4% [10.2–10.7%]) for frequent mental distress; and 20.8% (9.2% [9.0–9.3%] vs. 7.6% [7.4–7.8%]) for frequent activity limitation.

**Fig. 2:**
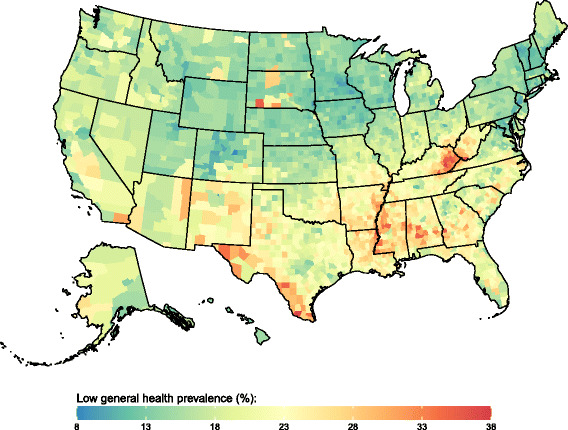
Low general health prevalence, 2012

**Fig. 3:**
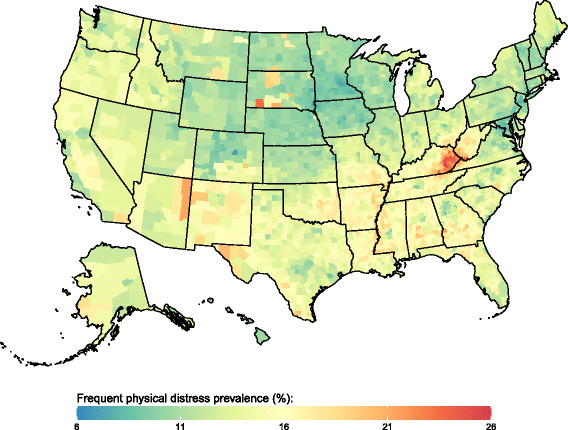
Frequent physical distress prevalence, 2012

**Fig. 4:**
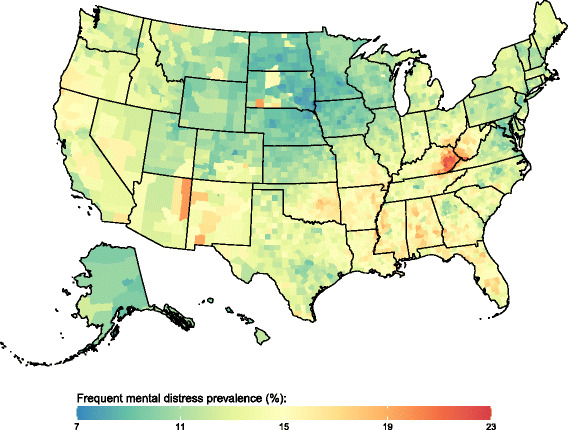
Frequent mental distress prevalence, 2012

**Fig. 5:**
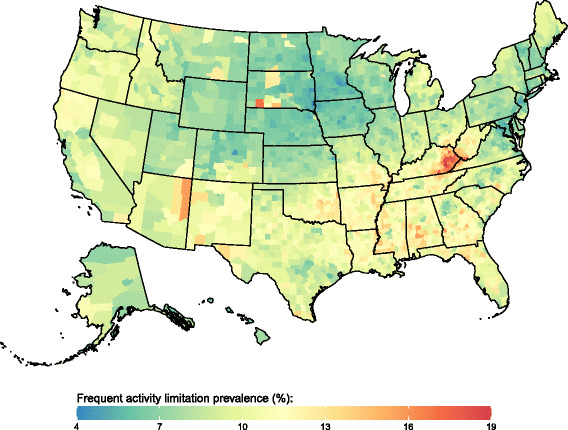
Frequent activity limitation prevalence, 2012

There was significant variation in all four outcomes at the county level in all years. The standard deviation of county-level prevalence of low general health decreased somewhat between 1995 (5.4 percentage points) and 2012 (5.1 percentage points). The standard deviation of county-level prevalence of frequent physical distress, frequent mental distress, and frequent activity limitation increased over this same period (from 2.2 to 2.7, 2.0 to 2.4, and 1.9 to 2.2 percentage points, respectively). Counties with the lowest prevalence of low general health (Fig. 2) were located primarily in New England and north-western states stretching from Utah to Wisconsin. In contrast, counties with the highest prevalence of low general health were found in parts of South Dakota, eastern Kentucky and western West Virginia, along the Texas-Mexico border, along the southern half of the Mississippi river, and in southern Alabama. Spatial patterns were similar for frequent physical distress, frequent mental distress, and frequent activity limitation (Figs. 3, 4 and 5). Results for all counties and all years are reported in Additional file 1.

**Fig. 6:**
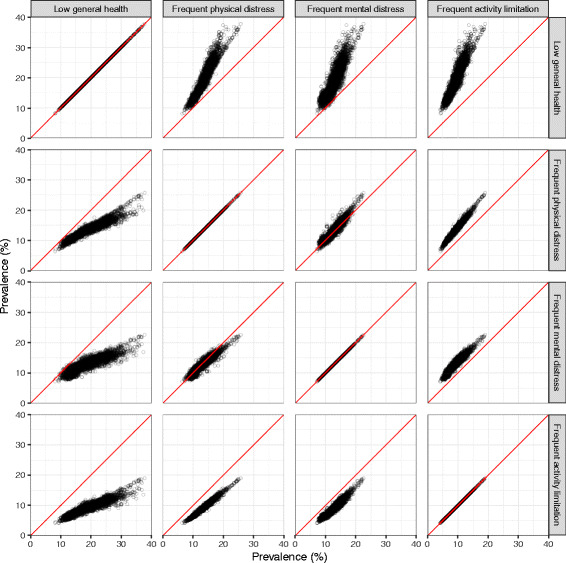
Comparison among self-reported health measures, 2012

Pairwise correlation coefficients between all four measures in 2012 were very high (Fig. 6), ranging from 0.88 (low general health and frequent mental distress) to 0.99 (frequent physical distress and frequent activity limitation). At the national level, the prevalence of low general health (17.5% [17.4–17.7%]) was highest and the prevalence of frequent activity limitation (8.4% [8.3–8.5%]) was lowest among the four measures, while prevalence of frequent mental distress (12.5% [12.3–12.7%]) and frequent physical distress (12.3% [12.2–12.5%]) were intermediate. This pattern was also observed at the county level, where the prevalence of low general health was nearly always the highest among the four measures, while the prevalence of frequent activity limitation was always the lowest. Levels of frequent physical and mental distress were generally similar within counties, except at the high end of the distribution: counties with very high prevalence of both typically had a slightly higher prevalence of frequent physical distress than frequent mental distress.

**Fig. 7:**
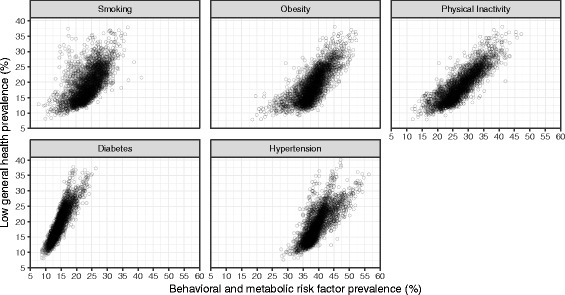
Relationship between low general health prevalence and prevalence of smoking, obesity, physical inactivity, diabetes, and hypertension

Low general health prevalence was positively correlated with the prevalence of behavioral and metabolic risk factors—0.63 for smoking, 0.76 for obesity, and 0.85 for physical inactivity—and with the prevalence of diabetes (0.90) and hypertension (0.78) (Fig. 7). Generally similar correlations were found between these variables and frequent physical distress, frequent mental distress, and frequent activity limitation (data not shown).

**Fig. 8:**
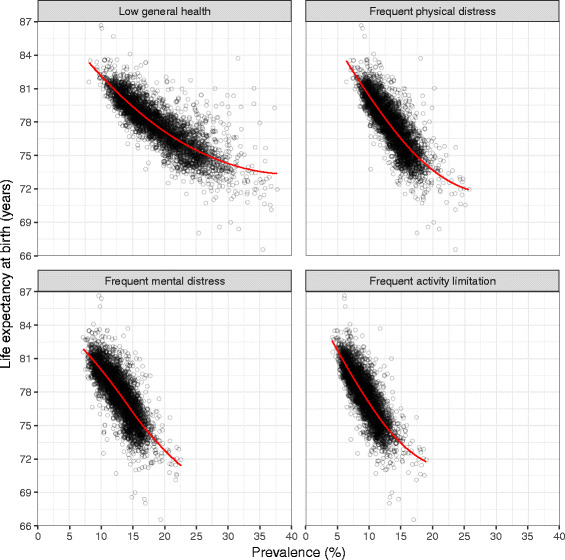
Relationship between life expectancy and self-reported health measures, 2012

Life expectancy was negatively correlated with all four measures (Fig. 8). The relationship between life expectancy and low general health was somewhat curvilinear, with a steeper decline in life expectancy as low general health prevalence moved from very low values to more moderate values, and a more moderate decline in life expectancy as low general health prevalence increased from moderate to high values. The relationship between life expectancy and the other three measures was closer to linear, but flattened somewhat among counties with very high prevalence of frequent physical distress, frequent mental distress, and frequent activity limitation.

**Fig. 9:**
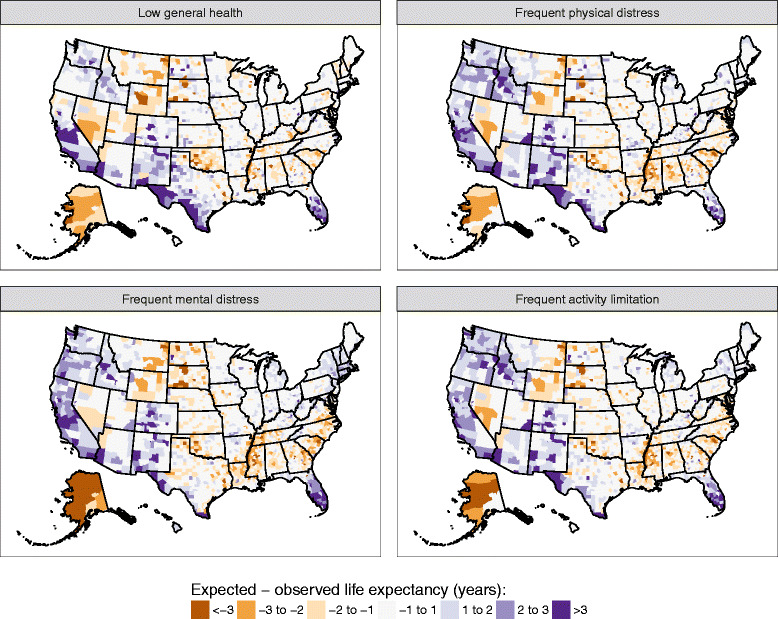
Gap between observed life expectancy and predicted life expectancy based on poor self-reported health prevalence, 2012

Figure 9 shows the difference between observed life expectancy and life expectancy predicted based on low general health prevalence, frequent physical distress, frequent mental distress, and frequent activity limitation. The spatial patterns are generally similar across these four measures. Counties in Western and Southwestern states (excluding Nevada) and in southern Florida tended to have higher life expectancy than average given their prevalence of poor self-reported health. In contrast, counties in Alaska, the Deep South (excluding Florida) and parts of Nevada and the upper Midwest and Great Plains regions tended to have lower life expectancy then average, given their prevalence of poor self-reported health.

Between 1995 and 2012, life expectancy increased in most counties (99.7%), while the prevalence of low general health, frequent physical distress, frequent mental distress, and frequent activity limitation also increased in most counties (88.1, 99.3, 98.3, and 99.2% of counties, respectively). There was a small negative correlation between change in life expectancy and change in the prevalence of frequent physical distress, frequent mental distress, and frequent activity limitation (Pearson correlation coefficients: −0.27, −0.23, and −0.22, respectively). When examined separately by sex, the correlations were again negative, but were generally larger among women (−0.25 to −0.35) than among men (−0.08 to −0.15). There was a weak positive correlation between changes in low general health prevalence and changes in life expectancy (0.09 overall; 0.12 for men and 0.06 for women).

## Discussion

This analysis found increasing rates and considerable geographic disparities in poor self-reported health within the US. These findings underscore the utility of local measurements of population health status and highlight the need for closer attention paid to geographic disparities in health outcomes.

The four measures considered—low general health, frequent physical distress, frequent mental distress, and frequent activity limitation—were highly correlated, though with important differences in some counties. Each of these measures were intended to capture a distinct facet of health: general health status (low general health), recent physical health (frequent physical distress), recent mental and emotional health (frequent mental distress), and recent day-to-day functioning (frequent activity limitation). The high correlations among these measures is likely a reflection of the close connections between different domains of health, though it may also indicate some overlap in the way each of the four healthy days questions were understood by respondents. Moreover, the close relationship between these four measures may also reflect shared determinants among different health domains, for example, socioeconomic factors.

Consistent with previous research at the individual level [10–12], we found that population-level prevalence of poor self-reported health (all four measures) was positively correlated with the prevalence of behavioral and metabolic risk factors. This may reflect a direct pathway from these risk factors to poorer health outcomes (e.g., smoking causing respiratory disease), but may also reflect individuals’ expectation of future health based on what they know about their own behaviors [5]. We also found a positive population-level relationship between poor self-reported health and chronic conditions such as diabetes and hypertension. These findings serve as an important external check on the validity of the self-reported health measures: all else equal, we expect poorer overall health when the prevalence of behavioral and metabolic risk factors or chronic conditions is high. However, the correlation among these variables may also reflect some common underlying determinants.

All four measures of poor self-reported health were strongly and negatively correlated with life expectancy at the county level. Nonetheless, life expectancy among counties with comparable levels of poor self-reported health often varied by multiple years, while the prevalence of poor self-reported health varied considerably among counties with similar life expectancy. This may reflect differences in non-fatal health outcomes: life expectancy captures only differences in survival, but not differences in health due to disabling but non-fatal conditions. However, this may also reflect differences in how respondents understand and respond to the healthy days questions, e.g., different understanding of what constitutes “good” health or a “healthy” day [33, 34]. Further research is required in the US to disentangle the extent to which geographic (or other) disparities in self-rated health reflect true disparities in health status.

Consistent with other studies utilizing BRFSS data, our analysis found that rates of poor self-reported health have increased at the national level as well as in most counties between 1995 and 2012 [16]. Over this same period, however, life expectancy has also increased nationally and in most counties (Laura Dwyer-Lindgren, Amelia Bertozzi-Villa, Rebecca W Stubbs, Chloe Morozoff, Johan P Mackenbach, Frank J van Lenthe, Ali H Mokdad, and Christopher JL Murray: Inequalities in life expectancy among US counties, 1980 to 2014: Temporal trends and key drivers., forthcoming). While changes in life expectancy were negatively correlated with changes in most of the self-reported health measures considered (i.e., the counties with smaller increases in life expectancy tended to have larger increases in poor self-reported health), this relationship is relatively weak. Additionally, comparative studies have highlighted differential trends in poor self-reported health among various US surveys [35]. Further research is needed to identify what is driving changes in poor self-reported health in the US and to identify to what extent these trends reflect true changes in underlying health status.

This study has a number of limitations. Survey response rates to the BRFSS are low and item non-response is also a concern. In both cases, missingness is not at random. Although we use post-stratification to explicitly account for factors such as education that are known to be related to both the likelihood of responding and the likelihood of reporting poor self-reported health, it is still possible that differential non-response biases our results. Moreover, the BRFSS, a telephone survey, excludes individuals with no phone and, prior to 2011, excluded individuals with only a cell phone. We have attempted to correct the latter issue, but some non-coverage bias may remain. The data sources we used for populations counts for post-stratification and for covariates for the small area models may also be subject to error. The small area model smooths both spatially and temporally; while this allows us to produce more precise estimates than otherwise possible, the model may in some cases over-smooth and thus underestimate variation in self-reported health. Finally, the BRFSS data used in this analysis were also used for generating the estimates of smoking, obesity, physical inactivity, diabetes, and hypertension prevalence and the correlation between the self-reported health measures and these risk factors may be somewhat higher in this analysis than they would be if these measures were based on independent data sources.

## Conclusions

Our findings revealed large disparities in the prevalence of poor self-reported health among counties in the US. Poor self-reported health was positively correlated with risk factor prevalence and prevalence of chronic health conditions and negatively correlated with life expectancy at the county level. Local information on health outcomes should be used by policymakers and health professionals to identify communities that are lagging behind, to evaluate the impact of policies and programs, and to monitor geographic inequalities.

## Additional file

Additional file 1: Prevalence of low general health, frequent physical distress, frequent mental distress, and frequent activity by county, year, and sex. (XLSX 30945 kb)
